# On Entanglement-Assisted Multistatic Radar Techniques

**DOI:** 10.3390/e24070990

**Published:** 2022-07-17

**Authors:** Ivan B. Djordjevic

**Affiliations:** Department of Electrical and Computer Engineering, University of Arizona, 1230 E. Speedway Blvd., Tucson, AZ 85721, USA; ivan@arizona.edu; Tel.: +1-520-626-5119

**Keywords:** entanglement, radars, quantum sensing, quantum radars, entanglement-assisted detection

## Abstract

Entanglement-based quantum sensors have much better sensitivity than corresponding classical sensors in a noisy and lossy regime. In our recent paper, we showed that the entanglement-assisted (EA) joint monostatic–bistatic quantum radar performs much better than conventional radars. Here, we propose an entanglement-assisted (EA) multistatic radar that significantly outperforms EA bistatic, coherent state-based quantum, and classical radars. The proposed EA multistatic radar employs multiple entangled transmitters performing transmit-side optical phase conjugation, multiple coherent detection-based receivers serving as EA detectors, and a joint detector.

## 1. Introduction

The entanglement represents a relevant quantum information feature [1,2,3,4,5,6,7,8,9,10] that can help us to (1) beat the classical sensor sensitivity [2,6,7,8], (2) provide unconditional security [4,5,6,10], and (3) beat corresponding classical channel capacities [1,6,7]. Additionally, we can use the pre-shared entanglement to implement distributed quantum sensing [6,8] and enable distributed and secure quantum information processing and computing [11].

Quantum radars can potentially outperform classical radars in terms of detection probability in a low-signal-to-noise-ratio (SNR) regime and range estimation. The main idea behind quantum radar research is to overcome the quantum limit of classical sensors and radars [6,8,9,12,13,14,15,16,17]. Even though quantum radars are significantly more challenging for practical implementation compared to classical ones, they still have several advantages, including improved receiver sensitivity, improved target detection probability, particularly in the low-SNR region (in comparison with classical radar signals, the quantum radar signals are much more difficult to intercept), improved quality of synthetic-aperture radar (SAR) imaging, improved detection of microwave photons through clouds and fog, improved resilience to jamming, and potentially higher cross-section area [16]. In recent years, the following quantum radar techniques emerged: (i) interferometric radar [5,16], (ii) quantum radars based on quantum illumination technique introduced by Lloyd [12] and discussed in [13,14], and (iii) the quantum enhanced noise radar technique [18]. Readers interested in learning more about various quantum radar techniques are referred to [6,15,16,17,18,19,20,21,22,23]. Various multistatic multiantenna backscattering communication scenarios were discussed in [24,25].

**Figure 1:**
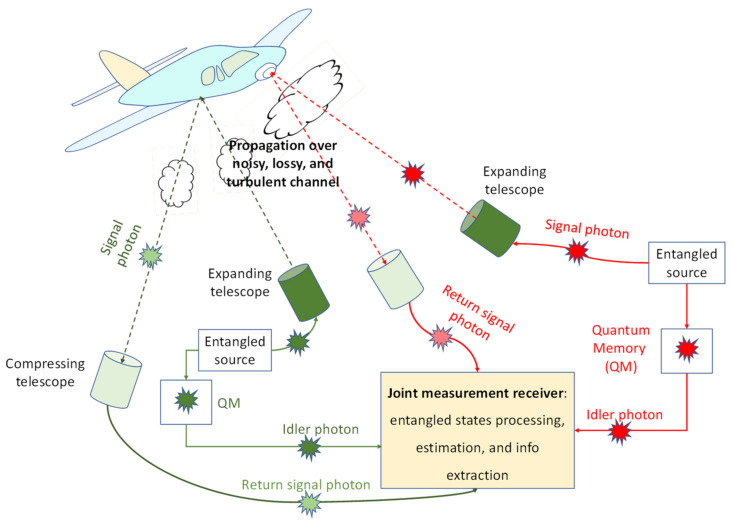
Illustrating the proposed EA multistatic 2 × 2 quantum radar concept. Multiple expanding telescopes are used so that different portions of the target can be illuminated. The entangled sources at different transmitters are mutually entangled. The forward scattered beams are collected by compressing telescopes and detected by EA receivers. To reduce overall complexity, the transmitter-side OPC is used, allowing integration of SPDC and OPC modules on a single chip. The EA receivers are composed of balanced homodyne detectors. The idler photons are stored in quantum memories and used as inputs to the balanced detectors once the presence of the return probes is detected. The other inputs to balanced detectors are return probes.

Here, we propose an entanglement-assisted (EA) multistatic quantum radar detection technique with the operational principle provided in Figure 1. We can interpret the proposed quantum radar technique as a generalization of the EA joint bistatic–monostatic scheme we proposed in [17]. Compared to the previous scheme, the proposed EA multistatic radar scheme has slightly higher complexity but much better performance and flexibility. The proposed multistatic radar scheme employs multiple entangled transmitters and multiple coherent detection-based receivers (see Figure 1), while the previous scheme only employed a single transmitter and monostatic and bistatic receivers. In this scheme, the phase-sensitive quantum correlation was exploited on the receiver sides with the goal of improving the overall detection probability of the target. Moreover, to increase the overall SNR, the spatial MIMO concept was used. Compared to the reflected and forward scattered components available in joint monostatic and bistatic radars, which are correlated and, as such, do not provide full spatial diversity, by using multiple transmitters that are properly separated in space, we can ensure statistical independence of different optical paths, thus achieving full diversity order in the proposed multistatic radar concept. This leads to improvements in both diversity order and array gain compared to the joint bistatic–monostatic scheme we studied in [17]. The introduced EA multistatic radar technique significantly outperforms the EA bistatic radar [9], EA joint bistatic–monostatic radar [17], coherent state-based quantum radars [26], and classical radars [27]. Additional details of the proposed multistatic radar technique are provided in Section 3. To study the performance of the proposed EA multistatic radar detection scheme, we modeled the scattered mode channels as noisy and lossy Bosonic channels, assuming imperfect entanglement distribution.

The organization of the paper is provided in the remainder of this section. For completeness of presentation, the EA bistatic radar technique [9] is described in Section 2, which is provided here for reference. The EA multistatic radar technique introduced here employs the optical phase conjugation (OPC) on the transmitter side and coherent detection on the receiver sides, as described in detail in Section 3. The forward scattered signal mode channels are modeled as noisy and lossy Bosonic channels. The idler channels are considered imperfect and modeled as less noisy and less lossy Bosonic channels compared to the signal mode channels. We study the detection probability performance of the proposed EA multistatic radar in Section 4, where we compare its performance against the EA bistatic radar, EA joint bistatic–monostatic radar, coherent state-based quantum radar, and classical radar detection techniques. In Section 5, some important concluding remarks are provided.

## 2. Entanglement-Assisted Bistatic Radar Technique

**Figure 2:**
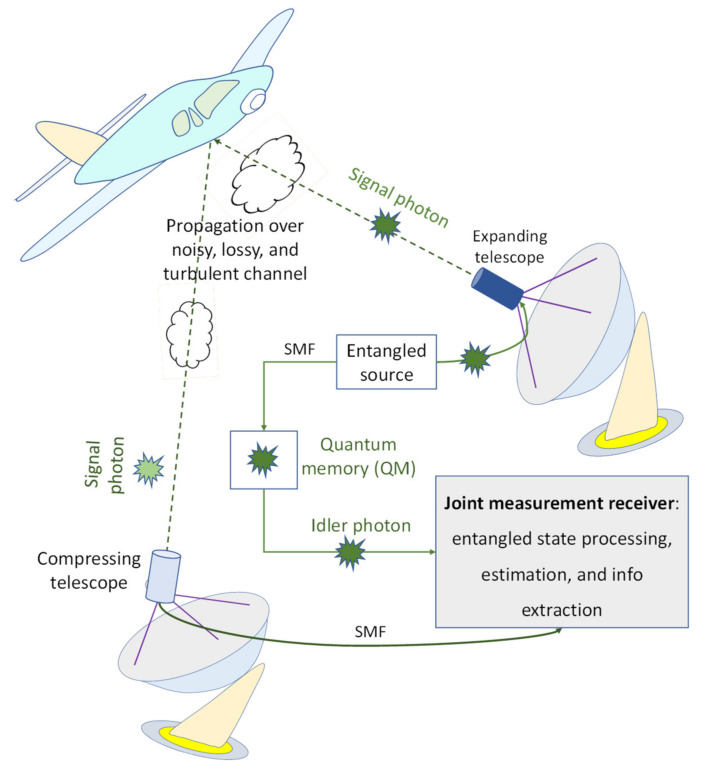
The EA bistatic quantum radar concept. The signal mode is directed toward the target with the help of the wide field of view of the expanding telescope. The return probe signal is collected by the compressing telescope and directed toward the EA detector, which is composed of the OPC module and a homodyne balanced detector. The idler photon is stored in quantum memory and used as the second balanced detector input once the presence of the return probe is detected.

Here, we briefly describe the EA bistatic radar concept we introduced in [9], illustrated in Figure 2, which is used as a referent case. This scheme is based on Gaussian states that are generated by utilizing the continuous-wave spontaneous parametric down-conversion (SPDC) process. As shown in Figure 2, the SPDC-based entangled source is employed on the transmitter side to generate a phase-sensitive quantum correlated signal photon (probe) and idler photon, which is used for the local reference. The expanding telescope directs the signal photon toward the target, while the transmitter–target–receiver channel is considered to be noisy and lossy, experiencing atmospheric turbulence. For better target detection, the expanding telescope should have a wide field of view. The forward scattered signal photon is collected by the compressing telescope and detected by remote bistatic radar’s receiver. The OPC takes place on the receiver side. The receiver is based on a balanced coherent detection with one input being the radar return and the second input being the idler. The idler is stored in a quantum memory, and it is used once the presence of the radar return is detected. In order to improve the receiver sensitivity, the existing phase-sensitive quantum correlation between the radar return signal photon and idler photon is utilized on the receiver side. Please refer to [9] for additional details.

Both the optical parametric amplifier (OPA)-based receiver and the OPC receiver can be used as EA receivers. Because the OPC receiver performs better than the OPA receiver [2,6,7], in order to simplify overall design, we introduce an EA multistatic target detection technique employing the transmitter-side OPC and classical coherent detection on the receiver sides, as described in the next section.

## 3. Proposed Entanglement-Assisted Multistatic Radar Technique

Here we describe our proposed EA multistatic radar detection technique, depicted in Figure 1, where a 2 × 2 MIMO concept is applied for illustrative purposes. As shown in Figure 1, the wideband entangled sources generate entangled pair of photons, with each pair containing signal and idler photons. The idler photons are stored in the receiver’s quantum memories (QMs). Given that QMs are not widely available, properly designed optical delay lines can be used instead. On the other hand, the signal photons are directed toward the target (by expanding telescopes) through a noisy and lossy Bosonic channel exhibiting atmospheric turbulence effects. The forward scattered photons are detected by the EA radar receivers. The spatial MIMO concept is utilized to improve tolerance to turbulence effects and increase the probability of target detection. The entangled transmitters are properly separated in such a way that the corresponding transmitter–target–forward scattered channels are statistically independent, allowing full diversity to be achieved. Multiple expanding telescopes per transmitter are used so that different portions of the target can be illuminated. Alternatively, multiple apertures per single telescope can be used instead. The entangled sources are based on the continuous-wave SPDC process, generating the signal–idler photon pairs. The SPDC source is an entangled broadband source containing *D* = *WT_m_* i.i.d. signal–idler photon pairs, where *W* represents the phase-matching bandwidth of the SPDC process, and we use *T_m_* to denote the measurement interval duration. Each signal–idler photon pair represents a two-mode squeezed vacuum (TMSV) state, with the corresponding representation in Fock basis given by

(1) $${|\psi \rangle }_{s,i}=\frac{1}{\sqrt{N_{s}+1}}\sum_{n=0}^{\infty }{\left(\frac{N_{s}}{N_{s}+1}\right)}^{n/2}{|n\rangle }_{s}{|n\rangle }_{i},$$

where $N_{s}=\langle {\hat{a}}_{s}^{†}{\hat{a}}_{s}\rangle =\langle {\hat{a}}_{i}^{†}{\hat{a}}_{i}\rangle$ is the mean photon number/mode. The corresponding signal and idler creation (annihilation) operators are respectively denoted by ${\hat{a}}_{s}^{†}\;\;({\hat{a}}_{s}^{})$ and ${\hat{a}}_{i}^{†}\;\;({\hat{a}}_{i}^{}).$ The phase-sensitive cross-correlation (PSCC) coefficient, defined as $\langle {\hat{a}}_{s}{\hat{a}}_{i}\rangle =\sqrt{N_{s}(N_{s}+1)}$, describes the signal–idler entanglement. The Wigner covariance matrix of the pure maximally entangled zero-mean Gaussian TMSV state is expressed as follows [17,28]:

(2) $$\Sigma_{TMSV}=\left[\begin{array}{cc} \left(2N_{s}+1\right)1 & 2\sqrt{N_{s}\left(N_{s}+1\right)}Z\\ 2\sqrt{N_{s}\left(N_{s}+1\right)}Z & \left(2N_{s}^{}+1\right)1 \end{array}\right],$$

where **1** is the identity matrix, and ***Z*** denotes the Pauli *Z*-matrix (diag(1, −1)). Clearly, when *N_s_* << 1, which is commonly referred to as the *low-brightness regime*, the PSCC coefficient is $\langle {\hat{a}}_{s}{\hat{a}}_{i}\rangle \approx \sqrt{N_{s}}$; compared to the corresponding classical limit *N_s_*, we have $\sqrt{N_{s}}\gg N_{s}$.

**Figure 3:**
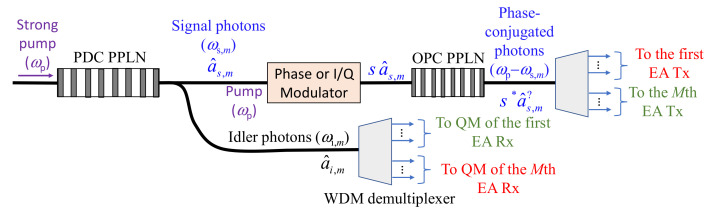
The integrated multistatic broadband EA transmitter (potentially based on LiNbO_3_ technology) with transmitter-side OPC. The phase or I/Q modulator is optional. Here, it is used to estimate the random phase shift introduced by the channel and to cancel it. We use *s* to denote a phase (or I/Q) modulator-induced signal constellation point, which, for Q-ary PSK, *s* is simply exp(j*θ_q_*), wherein *θ_q_* = *q*2π/*Q*; *q* = 0, 1, …, *Q* − 1. The following notation is used in the figure: QM, quantum memory; PPLN, periodically poled LiNbO_3_ waveguide; PDC, parametric down-conversion; OPC, optical phase conjugation; WDM, wavelength-division multiplexing.

To simplify the transceiver design and reduce the system cost, we apply the transmitter-side OPC so that classical balanced coherent detectors can be utilized as the EA detectors. Furthermore, we propose using a single broadband entangled source combined with a WDM demultiplexer as the common source for all transmitters, as illustrated in Figure 3. First, the periodically poled LiNbO_3_ (PPLN) waveguide serves as the SPDC source, which generates a large number of signal–photon pairs, wherein only the *m*-th signal-photon pair is illustrated in Figure 3. Signal and idler photons get separated by properly designed Y-junction. Idler photons get further separated by the WDM demultiplexer whose outputs are directed toward the QMs of corresponding EA receivers. On the other hand, all signal photons get simultaneously modulated by a training sequence known to all EA receivers, imposed by a Q-ary PSK modulator. This sequence is used to estimate the phase shift introduced by the target and channel. The common sequence is also used to determine the target range more precisely, by applying the cross-correlation method. The second PPLN waveguide is used to perform the OPC by employing the difference frequency generation (DFG) process, in which the *m*-th signal photon at angular frequency *ω*_s,*m*_ interacts with the pump photon *ω*_p_ to get the phase-conjugated (PC) photon at radial frequency *ω*_p_–*ω*_s,_*_m_*. The use of PPLN crystals to perform the OPC of WDM signals has already been demonstrated in classical communications [29]. We then use the WDM demultiplexer to demultiplex the signal photons to be used in multistatic transmitters, as depicted in Figure 1. We now provide an example corresponding to Figure 1, where the strong pump at *λ*_p_ = 780 nm is used. Through the SPDC process (with the help of the first PPLN waveguide), we can generate signal–idler pairs: (i) the idler photon 1 at wavelength *λ*_i,1_ = 1536 nm–the signal photon 1 at wavelength *λ*_s,1_ = 1584.8 nm; (ii) the idler photon 2 at wavelength *λ*_i,2_ = 1540 nm–the signal photon 2 at wavelength *λ*_s,2_ = 1580.5 nm. Through the DFG process (with the help of the second PPLN waveguide), the signal photon 1 (2) interacts with the pump photon to generate the phase-conjugated signal photon at wavelength *λ*_s,1,PC_ = 1536 nm (*λ*_s,2,PC_ = 1540 nm), which is the same wavelength as the idler photon 1 (2) wavelength.

**Figure 4:**
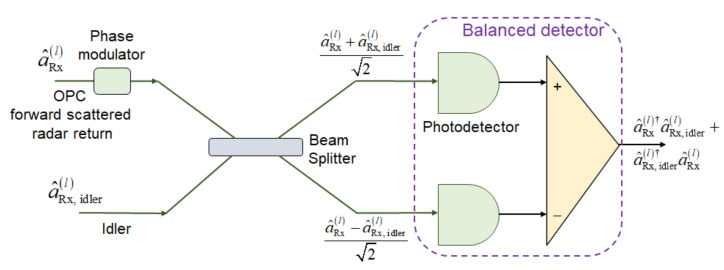
EA receiver corresponding to the *l*-th forward scattered component based on homodyne balanced detection. The receiver-side phase modulator is used to detect either the in-phase or the quadrature component of the corresponding PC signal. Without loss of generality, we set the photodiode responsivity to 1 A/W.

Given that the transmitter-side OPC is performed, we do not need to use the OPC-based EA receivers, but commercially available classical balanced coherent detectors can be used as the EA receivers instead, such as the one shown in Figure 4, thus reducing the overall system cost and complexity. This is particularly true when the number of receivers is much larger than the number of transmitters, whereby the number of required OPC modules can be reduced. Even when the number of receivers is comparable to the number of transmitters, it is still advantageous to place the OPC on the transmitter side because the SPDC and OPC modules can be integrated on the same chip, as discussed in the text related to the Figure 3.

The interaction between the forward scattered signal probe photon and the target can be described by a beam splitter of transmissivity *T*^(*l*)^, with one input being the probe and the other input being the thermal mode. Thus, we model the *m*-th radar transmitter-target–the *l*-*th* radar forward–scattered mode channel as a lossy and noisy Bosonic channel, which, for the transmitter-side OPC, is described by

(3) $${\hat{a}}_{Rx,m}^{\left(l\right)}\left(\varphi_{m}^{\left(l\right)}\right)=\sqrt{T_{m}^{\left(l\right)}}e^{-j\varphi_{m}^{\left(l\right)}}{\hat{a}}_{s,m}^{\left(l\right)†}+\sqrt{1-T_{m}^{\left(l\right)}}{\hat{a}}_{b}^{\left(l\right)},$$

where ${\hat{a}}_{b}^{\left(l\right)}$ is a thermal (background) state of the *l*-th scattered beam whose mean photon number is $\left(1-T_{m}^{\left(l\right)}\right)\langle {\hat{a}}_{b}^{\left(l\right)†}{\hat{a}}_{b}^{\left(l\right)}\rangle =N_{b}^{\left(l\right)}$.

The phase $\varphi_{m}^{\left(l\right)}$ has three components,

(4) $$\varphi_{m}^{\left(l\right)}=\theta_{\mathrm{mod}}+\vartheta_{m}^{\left(l\right)}+\phi_{m}^{\left(l\right)},$$

where *θ*_mod_ is the Q-ary PSK modulation phase, and $\vartheta_{m}^{\left(l\right)}$ is the deterministic phase shift introduced by the *l*-th forward scattered mode channel (originating from the *m*-th transmitter). Under the assumption that the distance between the receiver and the target in the forward scattered mode channel is $R_{m}^{\left(l\right)}$, the target-introduced phase shift is $\vartheta_{m}^{\left(l\right)}=k(r_{m}+R_{m}^{\left(l\right)})$, where *r_m_* is the distance between transmitter *m* and the target, while *k* denotes the wave number. Lastly, $\phi_{m}^{\left(l\right)}$ is the *l-*th scattered mode-introduced random phase shift. The phase modulator on the transmitter side, based on the Q-ary PSK, is employed to impose the sequence common for all transmitters, which is later used on the receiver sides to estimate the random phase shift. The common sequence can also be used to determine the delay between the signal and idler photons, by applying the cross-correlation method. Instead of sending a single pulse in each signaling interval, we can send a high-speed Q-ary PSK-modulated packet. Given that receivers know the transmitted sequence, they can apply the cross-correlation method, once the presence of the target is detected by the EA detector, to estimate the delay between signal and idler photons. The potential jammer will not know what the common sequence is and will need to apply the brute force approach.

The channel with idler mode is modeled as a less noisy and less lossy Bosonic channel compared to the scattered modes, which can be expressed as

(5) $${\hat{a}}_{\mathrm{Rx},\mathrm{idler}}^{\left(m\right)}=\sqrt{T_{m}^{\left(i\right)}}{\hat{a}}_{m}^{\left(i\right)}+\sqrt{1-T_{m}^{\left(i\right)}}{\hat{a}}_{b}^{\left(i\right)},$$

where $T_{m}^{\left(i\right)}$ denotes the idler channel transmissivity corresponding to the *m*-th transmitter, and ${\hat{a}}_{b}^{\left(i\right)}$ is the thermal (background) mode annihilation operator of the of the idler channel whose mean photon number is $\left(1-T_{m}^{\left(i\right)}\right)\langle {\hat{a}}_{b}^{\left(i\right)†}{\hat{a}}_{b}^{\left(i\right)}\rangle =N_{b}^{\left(i\right)}$. The relationship between the radar returned probe over the *l*-th scattered mode channel and retained reference (stored idler) corresponding to the *m*-th transmitter can be described by the Wigner covariance matrix as follows:

(6) $$\Sigma_{t}^{\left(m,l\right)}=\left[\begin{array}{cc} \left(2N_{s}+1\right)1 & 2\sqrt{T_{m}^{\left(l\right)}T_{m}^{\left(i\right)}N_{s}\left(N_{s}+1\right)}Z\delta_{1t}\\ 2\sqrt{T_{m}^{\left(l\right)}T_{m}^{\left(i\right)}N_{s}\left(N_{s}+1\right)}Z\delta_{1t} & \left(2N_{s}^{\left(m,l\right)}+1\right)1 \end{array}\right],$$

where $N_{s}^{\left(m,l\right)}=(T_{m}^{\left(i\right)}N_{s}+N_{b}^{\left(i\right)})T_{m}^{\left(l\right)}+N_{b}^{\left(l\right)}$, and subscript *t* is used as a target indicator. When the target is present, for *t* = 1, the antidiagonal terms, related to the phase-sensitive quantum correlation between the signal and idler, are nonzero. On the other hand, when the target is absent, for *t* = 0, the return signal contains only thermal (background) noise, and the covariance matrix is a diagonal one.

The photocurrent operator of the balanced detector (BD) (under the assumption that the photodiode responsivity is 1 A/W) for the EA detector corresponding to the *l*-th scattered mode channel and *m*-th transmitter, depicted in Figure 4, is given by

(7) $${\hat{i}}_{BD,m}^{\left(l\right)}={\left({\hat{a}}_{Rx.m}^{\left(l\right)}\right)}^{†}{\hat{a}}_{Rx,idler}^{\left(m\right)}+{\left({\hat{a}}_{Rx,idler}^{\left(m\right)}\right)}^{†}{\hat{a}}_{Rx,m}^{\left(l\right)},\;$$

For the receive side phase modulator shift being set to zero (∆*φ* = 0 rad), when the target is present (*t* = 1), the photocurrent operator expectation of the BD corresponding to the *l*-th scattered-mode channel and the *m*-th transmitter is obtained as

(8) $$\langle {\hat{i}}_{BD,m}^{\left(l\right)}\left(\Delta \varphi =0\right)\rangle =2\sqrt{T_{m}^{\left(i\right)}T_{m}^{\left(l\right)}N_{s}\left(N_{s}+1\right)}\cos\varphi_{m}^{\left(l\right)},\;$$

For the receiver-side phase modulator shift is set to ∆*φ* = −π/2 rad when the target is present (*t* = 1), the photocurrent operator expectation of the BD corresponding to the *l*-th scattered-mode channel and the *m*-th transmitter is obtained as

(9) $$\langle {\hat{i}}_{BD,m}^{\left(l\right)}\left(\Delta \varphi =-\pi /2\right)\rangle =2\sqrt{T_{m}^{\left(i\right)}T_{m}^{\left(l\right)}N_{s}\left(N_{s}+1\right)}\sin\varphi_{m}^{\left(l\right)}.$$

In order to determine the target range, we need to find the exact phase shift; to do so, both quadrature components are required. Specifically, from Equations (8) and (9), we can determine the overall phase as follows:

(10) $$\varphi_{m}^{\left(l\right)}=\tan^{-1}\left[\frac{\langle {\hat{i}}_{BD,m}^{\left(l\right)}\left(\Delta \varphi =-\pi /2\right)\rangle }{\langle {\hat{i}}_{BD,m}^{\left(l\right)}\left(\Delta \varphi =0\right)\rangle }\right].$$

Given that *θ*_mod_ is known by receivers, we determine the deterministic phase $\vartheta_{m}^{\left(l\right)}=k(r_{m}+R_{m}^{\left(l\right)})$ using Equation (4). By tapping the portion of the huge wavelength band of the SPDC process, we can use the classical heterodyne balanced detector, followed by the correlator, to determine the delay between the signal and idler photons, and this information can be used to adjust the variable optical delay line storing the idler photons. This delay is also related to the target range.

When the modulator shift of the receiver-side phase modulator is set to ∆φ = 0 rad, the variance of the photocurrent operator of the BD, defined as $Var\left({\hat{i}}_{BD,m}^{\left(l\right)}\right)=\langle {\left({\hat{i}}_{BD,m}^{\left(l\right)}\right)}^{2}\rangle -{\langle {\hat{i}}_{BD,m}^{\left(l\right)}\rangle }^{2}$, corresponding to the *m*-th transmitter and *l*-th scattered mode is given by

(11) $$Var\left({\hat{i}}_{BD,m}^{\left(l\right)}\right)=N_{m}^{\left(i\right)}N_{s,m}^{\left(l\right)}+\left(N_{m}^{\left(i\right)}+1\right)\left(N_{s,m}^{\left(l\right)}+1\right)\;+2N_{s}T_{m}^{\left(l\right)}T_{m}^{\left(i\right)}(N_{s}+1)\left[\cos\left(2\varphi_{m}^{\left(l\right)}\right)-2\cos^{2}\varphi_{m}^{\left(l\right)}\right],$$

where $N_{s,m}^{\left(l\right)}=(T_{m}^{\left(i\right)}N_{s}+N_{b}^{\left(i\right)})T_{m}^{\left(l\right)}+N_{b}^{\left(l\right)}$.

When the target is absent, the photocurrent operator expectation of the BD is zero, and, since *N*_i_ = *N*_s_, the corresponding variance is

(12) $$Var\left({\hat{i}}_{BD,t=0}^{\left(l\right)}\right)=N_{i}N_{b}^{\left(l\right)}+\left(N_{i}+1\right)\left(N_{b}^{\left(l\right)}+1\right)\;=N_{s}N_{b}^{\left(l\right)}+\left(N_{s}+1\right)\left(N_{b}^{\left(l\right)}+1\right).$$

In the problem of target detection, a priori probabilities are not known; hence, we need to employ the Neyman–Pearson criterion [27,30], in which we set the false alarm probability to the maximum tolerable value and maximize the detection probability of the target. Readers interested in getting more details on the Neyman–Pearson criterion in the context of quantum illumination are referred to [31,32,33].

The proposed EA multistatic radar *false alarm (FA) probability* is given by

(13) $$Q_{FA}=\frac{1}{2}erfc\left(\frac{t_{sh}}{\sqrt{N_{s}N_{b}+(N_{s}+1)(N_{b}+1)}}\right),$$

where *t_sh_* denotes the threshold derived from the set FA probability. The complementary error function in Equation (13) is defined by $erfc(x)=(2/\sqrt{\pi })\int_{x}^{\infty }\exp(-u^{2})du$.

When the maximum gain combining (MGC, see [34] for more details) is employed as the joint detection scheme (corresponding to all receivers), the *detection probability* of the target is determined by

(14) $$Q_{D}=\frac{1}{2}\mathrm{erfc}\left(\frac{t_{sh}-m_{v}}{\sqrt{\sum_{l,m}V_{m}^{\left(l\right)}}}\right),$$

where the overall mean value is given by

(15) $$m_{v}=2\sum_{l,m}\sqrt{T_{m}^{\left(l\right)}T_{m}^{\left(i\right)}N_{s}(N_{s}+1)},$$

where the summation is performed over all transmitters and scattered modes. The variance in Equation (15), originating from the *m*-th transmitter and *l*-th scattered mode, is given by

(16) $$V_{m}^{\left(l\right)}=N_{m}^{\left(i\right)}N_{s}^{\left(l\right)}+\left(N_{m}^{\left(i\right)}+1\right)\left(N_{s}^{\left(l\right)}+1\right)\;-2T_{m}^{\left(l\right)}T_{m}^{\left(i\right)}N_{s}(N_{s}+1).$$

Given that Gaussian states are used on the transmitter side, the corresponding probability density functions of the in-phase and quadrature components will have Gaussian distribution, which justifies the use of Gaussian assumptions in derivation of Equations (12) and (13). Derivation of the Wigner covariance matrix, given by Equation (6), as well as the BD operator expectation and variances, given by Equations (8), (11), and (12), is very similar to the author’s previous papers [9,28] and, as such, omitted.

## 4. Illustrative Results

**Figure 5:**
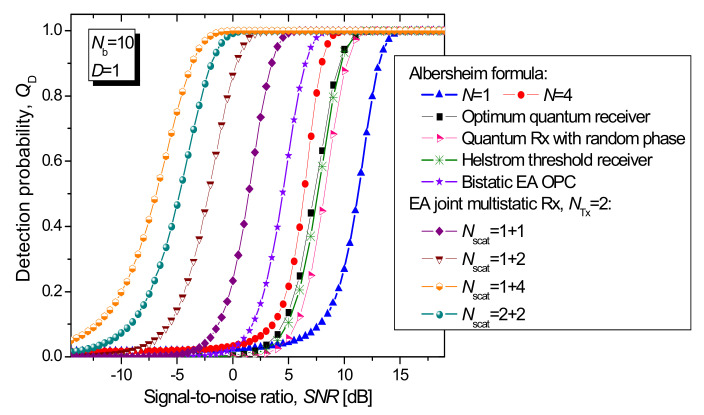
Detection probability of the target vs. SNR (dB) for different radar detection techniques with the average number of background photons set to *N_b_* = 10. The FA probability is set to *Q*_FA_ = 10^−6^. For comparison purposes, multistatic signal and idler channels are both assumed to be ideal. For the nonclassical target detection techniques, the signal-to-noise ratio is defined by *N_s_*/(2*N_b_* + 1), with *N*_s_ being the average number of signal photons.

Under the assumption that the signal and idler channels are ideal, by employing two transmitters (denoted as *N*_Tx_ = 2 in Figures below), and by setting the channel transmissivities to *T*^(*l*)^ = 1, in Figure 5, we compare the proposed EA multistatic target detection technique against various coherent states-based techniques (see [17] for details) and the EA detection technique for a bistatic radar, in terms of the detection probability of the target vs. SNR, with the average number of thermal photons being set to *N_b_* = 10 and system dimensionality *D* = 1, wherein the maximum tolerable FA probability that can be tolerated is set to *Q*_FA_ = 10^−6^.

The number of forward scattered components (as well as EA receivers), denoted as *N*_scat_ in Figure 5, is varied for the multistatic radar, with the first (second) component being the number of forward scattered components corresponding to the first (second) transmitter. The curves based on the classical Albersheim formula [27,35] are inserted with the number of samples set to *N* = 1 and 4. The following detection schemes based on coherent states are studied [17]: the optimum quantum detector, Helstrom threshold receiver, and random-phase quantum receiver. Clearly, the proposed EA multistatic radar technique significantly outperforms: the EA bistatic radar detection technique, various coherent state-based detection techniques, and the classical target detection technique.

**Figure 6:**
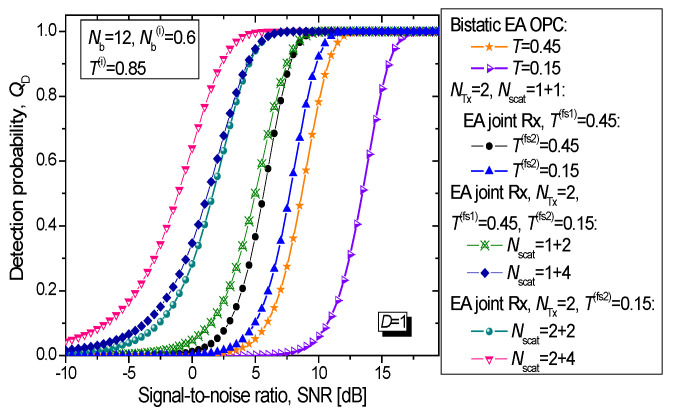
Target detection probability against SNR (dB) for proposed EA multistatic radar technique for idler channel transmissivities set to *T*^(i)^ = 0.85. The forward scattered mode channel transmissivity corresponding to transmitter 1 is set to *T*^(fs1)^ = 0.45. The forward scattered probe channel transmissivities related to transmitter 2 are varied within the range *T*^(fs2)^ ∈ {0.1, 0.45}. The *Q*_FA_ is set to 10^−6^.

In Figure 6, we study the target detection probability of the proposed EA multistatic radar technique (vs. SNR) assuming that the forward scattered probe channel transmissivities are different, wherein the average number of thermal photons is fixed to *N*_b_ = 12. For simplicity, we assume that all forward scattered channels corresponding to transmitter 1 are the same and equal to *T*^(fs1)^ = 0.45. On the other hand, we assume that all forward scattered channels corresponding to transmitter 2 are the same and can take one of two possible values *T*^(fs2)^ ∈ {0.15, 0.45}. The idler channels, being fiber-based, are assumed to be the same but less noisy and lossy (*N*_b_^(i)^ = 0.6 and *T*^(i)^ = 0.85). The EA multistatic radar technique for *T*^(fs1)^ = 0.45 and *T*^(fs2)^ = 0.15 outperformed the EA detector for the bistatic radar with transmissivity *T* = 0.45 by even 6.3 dB at *Q*_D_ = 0.95.

**Figure 7:**
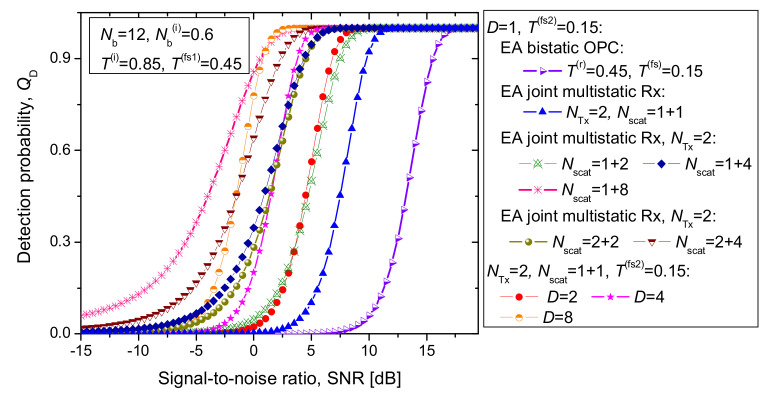
Target detection probability against SNR (dB) for proposed EA multistatic radar technique. The number of forward-scattered modes and system dimensionality were used as parameters. The idler channel transmissivities are fixed to *T*^(i)^ = 0.85. The forward scattered mode channel transmissivity corresponding to transmitter 1 (2) is fixed to *T*^(fs1)^ = 0.45 (*T*^(fs2)^ = 0.15). The *Q*_FA_ is set to 10^−6^.

In Figure 7, we set the system parameters similarly to in Figure 6 but varied the number of scattered components and system dimensionality *D*. As before, the EA multistatic scheme with two transmitters and two scattered components significantly outperformed the corresponding EA bistatic scheme (for *D* = 1). For high detection probabilities, the EA multistatic scheme with two scattered components corresponding to the first transmitter and two corresponding to the second performed comparably to the EA multistatic scheme with four scattered modes corresponding to the second transmitter and one corresponding to the first. When the number of scattered components was small, the increased system dimensionality could be used to improve the system performance. For *D* = 2 and the number of scattered components per transmitter being one, we could outperform the performance of the *D* = 1 scheme with the number of scattered components being 1 + 2. For a detection probability larger than 0.9, the *D* = 8 scheme with the number of scattered components being 1 + 1 outperformed the *D* = 1 scheme with the number of scattered components being 1 + 8. However, it performed worse for lower detection probabilities.

## 5. Conclusions

The EA multistatic quantum radar detection technique was proposed, employing the proposed integrated entangled source shared among multiple transmitters and performing the transmitter-side optical phase conjugation. EA receivers were based on classical homodyne detection schemes.

The proposed EA multistatic radar detection technique was evaluated against the bistatic radar EA detection scheme and various coherent state-based quantum detection schemes (the optimum quantum detector, Helstrom threshold detector, and random phase optimum quantum detector). We showed that the proposed EA multistatic target detection probability is significantly better than that of the corresponding bistatic radar EA detection technique, coherent state-based quantum detection techniques, and the classical radar detection schemes. When both scattered signal photon channels and idler channels are noisy and lossy, the proposed scheme significantly outperforms the EA bistatic radar scheme. When the number of scattered components is low, to improve detection probability performance, the system dimensionality should be increased.

## Data Availability

Not applicable.
